# Acute malnutrition and its determinants of preschool children in Bangladesh: gender differentiation

**DOI:** 10.1186/s12887-021-03033-z

**Published:** 2021-12-13

**Authors:** Md. Reazul Karim, Abu Sayed Md. Al Mamun, Md. Masud Rana, Rashidul Alam Mahumud, Nurun Naher Shoma, Dhiman Dutt, Premananda Bharati, Md. Golam Hossain

**Affiliations:** 1 Department of Statistics, University of Rajshahi, Rajshahi, 6205 Bangladesh; 2 DASCOH Foundation, Lutheren Mission Complex, Dingadoba, Rajpara, Rajshahi, 6201 Bangladesh; 3 NHMRC Clinical Trials Centre, Faculty of Medicine and Health, The University of Sydney, Camperdown, NSW 2006 Australia; 4 Swiss Red Cross, House# 35, Road # 117, Gulshan-1, Dhaka, 1212 Bangladesh; 5 Biological Anthropology, Indian Statistical Institute, 203 BT Road, Kolkata, West Bengal 700 108 India

**Keywords:** Acute malnutrition, Associated factors, Preschool children, Gender differentiation, Logistic regression model

## Abstract

**Background:**

Children acute malnutrition (AM) is a global public health concern, especially in low and middle income countries. AM is associated with multiple physiological vulnerabilities, including immune dysfunction, enteric barrier disruption, gut microbiome dysbiosis, and essential nutrient deficits. This study aimed to determine the prevalence of AM and its associated factors among preschool children in Rajshahi district, Bangladesh.

**Methods:**

This cross-sectional study was conducted from October to December, 2016. Children acute malnutrition was assessed using mid-upper arm circumference. Multiple binary logistic regression analyses were employed to determine the associated factors after adjusting the effect of independent factors of children AM.

**Result:**

The prevalence of AM amongst preschool children was 8.7%, among them 2.2 and 6.5% were severe acute malnutrition and moderate acute malnutrition, respectively. Z-proportional test demonstrated that the difference in AM between girls (11.6) and boys (5.9%) was significant (*p* < 0.05). Children AM was associated with being: (i) children aged 6–23 months (aOR = 2.29, 95% CI: 1.20–4.37; *p* < 0.05), (ii) early childbearing mothers’ (age < 20 years) children (aOR = 3.06, 95% CI: 1.08–8.66; *p* < 0.05), (iii) children living in poor family (aOR = 3.08, 95% CI: 1.11–8.12; *p* < 0.05), (iv) children living in unhygienic latrine households (aOR = 2.81, 95% CI: 1.52–5.09; *p* < 0.01), (v) Hindu or other religion children (aOR = 0.42, 95% CI: 0.19–0.92; *p* < 0.05).

**Conclusion:**

The prevalence of AM was high among these preschool children. Some modifiable factors were associated with AM of preschool children. Interventions addressing social mobilization and food security could be an effective way to prevent acute malnutrition among children in Bangladesh.

## Introduction

Acute malnutrition is one of the leading public health concerns globally, including in low- and middle-income countries (LMICs) [1]. Approximately 52 million children (< 5 years) are suffered from AM worldwide, among them 36 million of children who have experienced of AM in Asia [2]. In addition, more than 17 million children are suffered from severe acute malnutrition (SAM). Children with SAM are nine times more likely to die than well-nourished children [3]. AM in compare to well-nourish, have a three-fold increased risk of mortality, increases the risk of stunted growth, impaired cognitive development [4], non-communicable diseases in adulthood and deaths from infectious diseases such as diarrhea, pneumonia and measles. Moderate acute malnutrition (MAM) is more prevalent than severe acute malnutrition (SAM), and affects approximately 64% of all those categorized as having AM [1]. AM has affects more than 50 million under-five children, causing 8.0% of child deaths globally each year [5, 6]. The findings show that more than 400,000 child deaths can be prevented each year by adequate identification and proper management of AM [7]. Emerging factors (e.g., food insecurity, inadequate balance diets) are significantly contributed to AM [5]. Furthermore, AM leads to bilateral pitting edema or sudden weight loss, childhood comorbid conditions, and inadequate physical and psychological developments [8].

Several methods can be employed to measure AM. For example, AM can be defined based on bilateral pitting edema or wasting (low mid upper arm circumference (MUAC)) or low weight-for-height measurements [9]. The World Health Organization (WHO) has defined AM to measure the children nutritional status if SAM (MUAC < 115 mm) and MAM (115 mm ≥ MUAC < 125 mm) for children ages 6–59 months or weight-for-height (− 2 standard deviations, SD < Z-scores) of the WHO reference population [10]. MUAC is a widely used rapid nutritional assessment approach based on the assumption that it closely related to muscle mass to identify SAM in children [11]. In practice, MUAC is used on a large scale as a single tool for detecting SAM because it is cheap, and community health workers can learn to use it effectively with minimal training. The tool is also well accepted by children, due to the simplicity of its measurement [11, 12]. During these calamities, childhood illnesses like diarrhea and pneumonia are occurred [13], and in large extent war and pandemic. Initially, SAM was seen as a problem primarily in emergency contexts, but globally there has been growing recognition of its extent in nonemergency situations [14]. The majority of SAM cases occur in developing countries not affected by any emergencies.

Moderate and severe malnutrition account for 40% to 50% of all deaths in children under-5 years of age [15]. SAM is one of the top three nutrition-related causes of death in children under five. Malnutrition contributes 8 million deaths in children under 5 years of age worldwide [16]. SAM associated with 1–2 million preventable child deaths each year [17, 18]. Survivors of acute malnourished children are at increased risk of developing stunting and various diseases, disorders, poor educational performance, and low productive life [19]. Children who are at high risk of SAM are usually in risk of associated morbidity and negative effects on their growth and cognitive development later in life [20]. The underlying determinants of malnutrition in South Asia are manifold and in some respect multiplicative [21]. For example, high population density combines with poverty, inadequate food access, lack of hygiene and safe water, and other factors to keep the prevalence of malnutrition virtually unchanged. Diets deficient both in macronutrients and micronutrients in combination with a high burden of infection are the main underlying causes of child AM [22].

Gender refers to the social relationships between males and females in terms of their roles, behaviors, activities, attributes and opportunities, and which are based on different levels of power. It is socially constructed and can change overtime gender inequality and discrimination faced by women and girls puts their health and well-being at risk [23]. Gender equality for women and girls are matter in nutrition because they have an important bearing on the three underlying determinants of nutrition: food security; care practices; and health [24]. In South Asia, increases in women’s status have a strong influence on both the long- and short-term nutritional status of children, leading to reductions in both stunting and wasting [25]. Gender inequality in nutrition is strongly associated with eating, food distribution and nutritional habits that influence women and/or men nutritional status. Women and girls are the main victim of the “food discrimination”, which results in chronic undernutrition and ill health [26]. Gender inequality can be a cause as well as an effect of hunger and malnutrition. Not surprisingly, higher levels of gender inequality are associated with higher levels of undernutrition, both acute and chronic undernutrition [27]. Gender inequality and malnutrition through their influence and interaction with health conditions affect the different aspects and dimensions of socioeconomic development [28].

Gender equality in nutrition is important due to failing to address nutrition for everyone, and particularly for women and children, undermines the success of all the SDGs. Improvements in nutritional equality in gender are important to achieving the Sustainable Development Goals (SDGs), ending hunger and improving nutrition remains a top priority for international development. It closely links some goals of SDG such as number 2 (‘End hunger, achieve food security and improved nutrition and promote sustainable agriculture) of the SDGs and number 5 is gender equality, with SDG Target 2.2 aiming to “End all forms of malnutrition” by 2030. It strives to contribute to goals by exploring the link between malnutrition and gender equality [29]. The global Sustainable Development Goals (SDGs) include a global target for 2025 aimed at reducing, and then maintaining, child wasting to below 5% [30].

Level of stunting among Bangladeshi children < 5 years declined from 51% in 2004 to 36% and underweight from 41% in 2007 to 33% [31]. But the decrease in wasting rate is not as expected, which is only from 17 to 14.3% over last decade. Approximately 3.1% (BDHS 2014) of under-5 children suffering from SAM only by weight-for-length or height z-score (WHZ) < − 3 criterion and estimated to be a total of 450,000. SAM is the most extreme and visible form of undernutrition and its prevention and treatment are critical to child survival and development. Almost 15% of South Asian children have AM. In India, approximately 28 million (15.5%) preschool children have wasting, with over 5 million children being severely wasted. Rates of AM are above 15% in Bangladeshi preschool children [32]. Many studies reveal that AM leads to more severe infection and higher case fatality in Bangladeshi children. The death rate among children hospitalized for SAM was as high as 15% in Bangladesh [33]. From the previous study review that AM among preschool children observer during emergency and nonemergency period, using MUAC to quick identified the status in Rajshahi district of Bangladesh for further recommendation.

Therefore, the aim of the study was to determine the prevalence and associated factors of AM among preschool children in Rajshahi district, Bangladesh. In addition, we wanted to find the difference in nutritional status between boys and girls.

The study was based on the following hypotheses:

- H_01_: Socio-economic and demographic factors have significantly associated with AM of preschool children.
- H_02_: Health related factors are significantly associated with AM of preschool children.
- H_03_: There is no significantly different in nutritional status between boys and girls.

## Methods

**Fig. 1:**
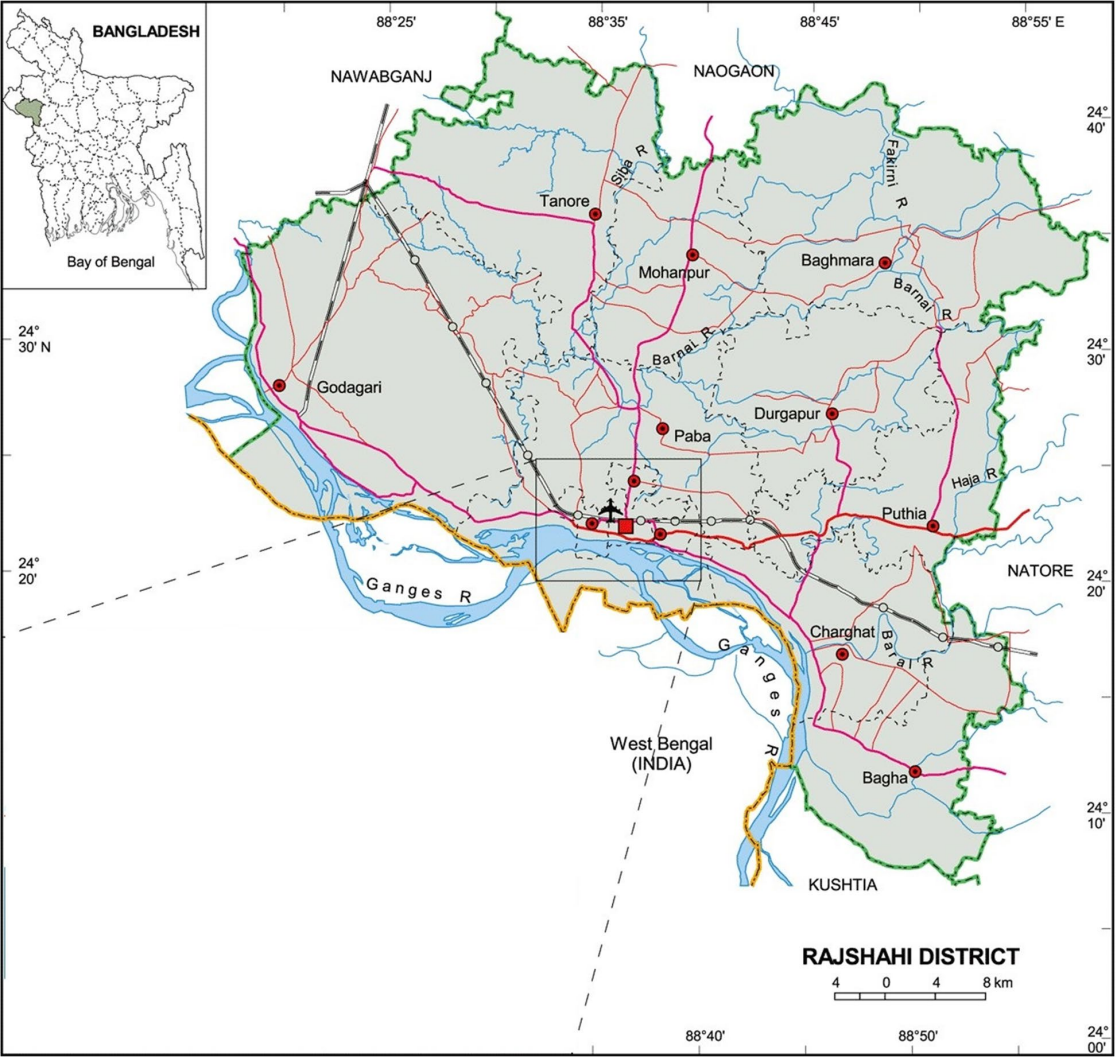
Map of Rajshahi district (source: Banglapedia)

The rural area of Rajshahi district was the study area and preschool (aged 6–59 months) were considered as population of this study. This cross sectional study was conducted from October to December, 2016. Rajshahi district located in the north-western part of Bangladesh and separated from India by a branch of Ganges River (Padma branch). The total area of the district is 2425.37 km^2^ and the population are 2,699,688 [34] and consists of 9 Upazilas, 71 Unions and 1587 villages (Fig. 1). Rajshahi is the one of the highest poverty zones in Bangladesh. Around 2.3% ethnic minority representative whom is most deprived population [34].

### Sample size determination

The following mathematical formula was used to determine the sample size for this study [35]:

1 $$n=\frac{z^2p\left(1-p\right)}{d^2},$$

Here, n = the required number of samples, z = 1.96 (95% CI), *p* = 0.15, the probability of prevalence of AM of preschool children in Bangladesh [32] and d = 0.05 (margin of error). The formula provided that 196 pre-school children was the minimum sample size for the study. However, initially, 540 pre-school children were considered.

### Sampling procedures

Multistage sampling technique was utilized to select samples covering all the population from all Upazilas (UP) of Rajshahi district. There are 9 UPs of Rajshahi district and in these UPs 232 Community Clinics (CCs) are running. In the first stage, 2 CCs had been selected from each UP by simple random sampling. A total of 18 (9 × 2) CCs were selected. In the second stage, 2 villages had been selected from each selected CC catchments areas by random sampling, total of 36 (18 × 2) villages were selected. In the third stage, 16 households had been selected from each selected village by random sampling those had at least one mother having at least one preschool children. The mothers and their children were identified by their identification number (holding number or listing). All information was got from respective UP. If there was more than one mother having pre-school children in same household, one mother with her child had been selected by lottery. If selected mother had more than one preschool child, last born child was considered. Since 36 individuals did not agree to participate in our study, consequently, a total of 540 preschool children with mean age 29.27 months were considered as a sample in the present study.

### Data collection procedure

A partially structured questionnaire which was duly pre-tested for collecting data from the respondents. We prepared the original questionnaire in English, and it was sent to five experts in health sciences for getting their opinions, suggestions and comments. We followed their suggestions to finalize the original questionnaire, and translated it to Bangla (mother tongue of Bangladeshi) for easy understandability. The present authors checked the meaning of Bangla questionnaire with original questionnaire. The first and third authors collected data from our selected households. Data was collected by using interview administrative method, the questionnaire was filled out by data collectors. A mini data collection was done from different study fields to observe whether there was any lacking or drawback in the questionnaire. After proper modification questionnaire was finalized and made ready for data collection. Before collecting data we discussed in detail the purpose of our study with guardians of selected children, and written consent had been taken from them. Completed questionnaires were checked on a daily basis for accuracy and completeness in the recording of responses. Editing and coding were done before data entry. Data were entered and analyzed using SPSS (IBM version 22). The presence of abnormal points in the data set can affect the interpretation of results. The present author checked the outlier of the data set.

### Outcome variable

Nutrition status was the dependent variable in this study, and it was measured by mid-upper arm circumference (MUAC). MUAC is a widely used rapid nutritional assessment to identify SAM in children for well accepted by them, due to the simplicity of its measurement [14]. In practice, MUAC is used on a large scale as a single tool for detecting SAM because it is cheap, and community health workers can learn to use it effectively with minimal training [11]. The mid upper arm circumference (MUAC) of children aged 6–59 months gives an indication of the degree of wasting or AM status. According to WHO (2006), the nutritional status of preschool children was classified as the cut-off points of MUAC such as: (i) SAM (MUAC< 115 mm), (ii) MAM (115 < MUAC< 125 mm) and (iii) normal nutrition (MUAC≥125 mm). Finally, we classified our sample into two groups; (i) AM ((SAM + MAM) if MUAC< 125 mm)); code 1 and, (ii) normal nutrition (MUAC≥125 mm); code 0 for further statistical analysis.

### Independent variables

The following socio-economic and demographic variables were used in this study as independent variables: Children’s age [(in months) 1: 6–24 month; 2: 25–59 month)]; Children’s gender (1: Boy; 2: Girl); Father’s education (1: Uneducated, 2: Primary, 3: Secondary, 4: Higher education); Mother’s education (1: Uneducated, 2: Primary, 3: Secondary, 4: Higher education); Father’s occupation (1: Farmer, 2: Labour, 3: Business, 4: Service holder); Mother’s occupation (1: Housewife, 2: Others); Mother’s age (1:< 20 year, 2:≥20–25 year; 3: 26–34 year, 4:≥35 year); Child birth weight (1: Normal birth weight ≥ 2500 g, 2: Low birth weight < 2500 g); Household average monthly income [(in Bangladeshi Taka) (1: < 5000 Taka, 2: ≥5000–9999 Taka, 3: ≥10,000 Taka)]; Hygienic having hygienic latrine (1: Yes, 2: No); Children received zinc treatment during diarrhea (1: Yes, 2: No); Religion (1: Islam, 2: Other); Having electricity in household (1: Yes, 2: No). These independent variables were selected on the basis of previous study/s [7, 33].

### Statistical analysis

Descriptive statistics was carried out to find the prevalence of AM (SAM + MAM), and Z-proportional test was used to find the difference in different categories of malnutrition between boys and girls children. Chi-square test was applied to find the association between selected socio-economic and demographic factors and child nutritional status. Multiple logistic regression model was utilized in this study to determine the effect of selected factors on children AM. Multicollinearity problems among independent variables in multiple logistic regression model was checked by the magnitude of standard error (SE), if SE value was less than 0.50, considered there was no evidence of multicollinearity problem [36]. Finally, the independent contribution of each factor was calculated by (individual Nagelkerke R^2^ value /total Nagelkerke R^2^ values) × 100 [37]. A statistical package, SPSS (Version IBM, 22) was used to carry out the entire analysis. A value of *p* < 0.05 was considered statistically significant in the analysis.

## Results

A total of 540 under-five children (age, 6–59 months) were recruited in this study, among them 50.6 and 49.4% were boys and girls respectively. 37.8 and 62.2% children were 6–23 and 24–59 months age respectively, with mean age 29.72 ± 14.59 month. 65.7% mothers were 20–29 years old. We observed that 90% children was born with normal weight, 42.8% children living in middle family (monthly income, 5000–9999 Taka), and 68.9% children living in house where hygienic latrine facility was available. More than 50% children did not receive zinc during diarrhea treatment. Still 18.9 and 14.1% fathers and mothers were uneducated in our study area, more than 40% fathers were farmers, most of the mothers were housewives (96.4%), and 89.4% were Muslim (Table 1). Chi-square test demonstrated that children’s age (*p* < 0.01), gender of children (*p* < 0.05), mother’s age (*p* < 0.01), child birth weight (*p* < 0.05), household monthly income (*p* < 0.01), household having hygienic latrine (*p* < 0.01), children receive zinc treatment during diarrhea (*p* < 0.05), religious (*p* < 0.01) and having electricity at household (*p* < 0.05) were significantly associated factors of nutritional status of pre-school children (Table 1). These variables factors considered as independent variables in multiple logistic regression analysis.

**Table 1:** Frequency distribution and association between nutritional status of preschool children and socio-demographic factors

Variable	Group	Nutritional status		Chi-square test-value	*p*- value
		MUAC ≤ 125 mm (Acute malnutrition)	MUAC > 125 mm (Normal nutrition)		
Children’s age (in month)	6–23, 204 (37.8%)	13.7%	86.3%	10.405	0.001
	24–59, 336 (62.2%)	5.7%	94.3%		
Gender of children	Boys 273 (50.6%)	5.9%	94.1%	5.616	0.018
	Girls 267 (49.4%)	11.6%	88.4%		
Father’s education	Uneducated 102 (18.9%)	8.8%	91.2%	0.006	1.00
	Primary 184 (34.1%)	8.7%	91.3%		
	Secondary 140 (25.9%)	8.6%	91.4%		
	Higher education 114 (21.1%)	8.8%	91.2%		
Mother’s education	Uneducated 76 (14.1%)	10.5%	89.5%	0.391	0.942
	Primary 121 (22.4%)	8.3%	91.7%		
	Secondary 252 (46.7%)	8.3%	91.7%		
	Higher education 91 (16.9%)	8.8%	91.7%		
Father’s occupation	Farmer 225 (41.7%)	11.6%	88.4%	4.521	0.210
	Labour 192 (35.6%)	5.7%	94.3%		
	Business 83 (15.4%)	8.4%	91.6%		
	Service holder 40 (7.4%)	7.5%	92.5%		
Mother’s age (in years)	< 20 year 41 (7.6%)	22.0%	78.0%	9.799	0.007
	≥20–29 year 355 (65.7%	7.6%	92.4%		
	≥30 year 144 (26.7%)	7.6%	92.4%		
Child birth weight	Normal birth weight ≥ 2500 g 486 (90.0%)	7.8%	92.2%	4.788	0.029
	Low birth weight < 2500 g 54 (10.0%)	16.7%	83.3%		
Household average monthly income (in Bangladeshi Taka)	< 5000 Taka 151 (28.0%)	14.6%	85.4%	10.411	0.005
	≥5000–9999 Taka 231 (42.8%)	7.8%	92.2%		
	≥10,000 Taka 158 (29.3%)	4.4%	95.6%		
Household having hygienic latrine	Yes 372 (68.9%)	5.9%	94.1%	11.711	0.001
	No 168 (31.1%)	14.9%	85.1%		
Children received zinc treatment during diarrhoea	Yes 263 (48.7%)	5.7%	94.3%	5.808	0.016
	No 277 (51.3%)	11.6%	88.4%		
Religion	Islam, 483 (89.4%)	7.5%	92.5%	9.002	0.003
	Other, 57 (10.6%)	19.3%	80.7%		
Having electricity in household	Yes, 438 (81.1%)	7.3%	92.7%	5.701	0.017
	No, 102 (18.9%)	14.7%	85.3%		

It was observed that the standard errors of all selected variables were less than 0.50, there was no evidence of multicollinearity problems among independent variables in multiple logistic regression model. After controlling the effect of other variables, multiple logistic regression model demonstrated that younger children (age, 6–23 months) had a 2.29-fold higher chance to get acute malnutrition than older children (age, 24–59 months) [aOR = 2.29, 95% CI: 1.20–4.37; *p* < 0.05]. Early child bearing mothers’ (age < 20 years) children were more likely to have acute malnutrition than older mothers’ (age > 30 years) children [aOR = 3.06, 95% CI: 1.08–8.66; *p* < 0.05]. Children living in poor family (monthly income< 5000 Taka) had more risk to get acute malnutrition than children living in rich family (monthly income≥10,000 Taka) [aOR = 3.08, 95% CI: 1.11–8.12; *p* < 0.05]. Children living a household did not have hygienic latrine were more likely to get acute malnutrition [aOR = 2.81, 95% CI: 1.52–5.09; *p* < 0.01] compared to their counterpart. Children living in Muslim family had 58.5% no risk to get acute malnutrition than Hindu or others religious children [aOR = 0.42, 95% CI: 0.19–0.92; *p* < 0.05]. The individual contribution of the significant influencing factors; children’s age, mother’s age, household monthly income, household having hygienic latrine and religion were 10.95, 14.96, 10.95, 15.33, 16.42% and (10.95%) respectively. Hosmer and Lemeshow test showed that our selected model was good fitted to the data [Chi-square value = 3.65; *p* > 0.05] (Table 2).

**Table 2:** Effects of socio-demographic factors on acute malnutrition of preschool children

Independent variables	Coefficient (β)	SE of (β)	*p*-value	Adjusted Odds Ratio (aOR)	95% CI of AOR	Individual contribution, (%)
**Children’s age (in month)**						14.96
6–23	0.83	0.33	0.013	2.29	1.20–4.37	
24–59®				1.00		
**Gender of children**						8.76
Boy	−0.54	0.34	0.120	0.59	0.36–1.15	
Girl®				1.00		
**Mother’s age (in year)**						10.95
< 20	1.12	0.53	0.036	3.06	1.08–8.66	
≥ 20–29	− 0.032	0.39	0.895	0.95	0.44–2.04	
≥ 30®				1.00		
**Child birth weight**						5.84
Normal birth weight ≥ 2500 g	−0.85	0.45	0.07	0.43	0.18–1.02	
Low birth weight < 2500 g®				1.00		
**Household monthly income (Taka)**						15.33
< 5000	1.10	0.41	0.030	3.08	1.11–8.12	
≥ 5000–9999	0.39	0.48	0.424	1.47	0.57–3.79	
≥ 10,000®				1.00		
**Household having hygienic latrine**						16.42
No	1.02	0.31	0.001	2.81	1.52–5.09	
Yes®				1.00		
**Children received zinc treatment during diarrhea**						9.12
No	0.57	0.35	0.101	1.77	0.89–3.51	
Yes®				1.00		
**Religion**						10.95
Islam	−0.88	0.41	0.030	0.42	0.19–0.92	
Other®				1.00		
**Household having electricity**						7.67
No	0.54	0.36	0.132	1.72	0.85–3.49	
Yes®				1.00		
**Constant**	−2.99	0.74	0.001	0.05		
**Goodness of fit**	Hosmer and Lemeshow Test		Chi-square value = 3.65		*p*-value = 0.887	

### Z-proportional test

It was noted that 8.7% pre-school children were suffering acute malnutrition, among them 2.2 and 6.5% were severe acute and moderate acute malnourished respectively, while still 91.3% children were normal weight. We observed that more number of girls (3.0%) were severe acute malnourished than boys (1.5%), however the difference was not significant (*p* > 0.05). More number of girls (8.6%) were moderate malnourished than boys (4.4%), the difference between girls and boys was significant (*p* < 0.05), consequently more number of girls (11.6%) were suffering from acute malnutrition than boys (5.9%), the difference was significant (*p* < 0.05). Significantly higher number of boys (94.1%) was normal weight than that of girls (88.4%) (*p* < 0.05) (Table 3).

**Table 3:** Prevalence and comparison of nutritional status between boy and girl

Type of nutritional status	N (%)	Boy, N (%)	Girl, N (%)	Z-proportional test value	*p*-value
MUAC< 115 mm (SAM) (severe acute malnutrition)	12 (2.2)	4 (1.5%)	8 (3.0%)	0.1569	0.8728
115 < MUAC ≤125 mm (MAM) (moderate acute malnutrition)	35 (6.5)	12 (4.4%)	23 (8.6%)	1.9827	0.0477
Acute malnutrition (SAM + MAM)	47 (8.7)	16 (5.9%)	31 (11.6)	2.3475	0.0188
> 125 mm (Normal)	493 (91.3)	257 (94.1%)	236 (88.4%)	2.4869	0 .0127
Total	540	273 (50.6%)	267 (49.4%)		

## Discussion

This study depicts the acute malnutrition among preschool children is 8.7%, which is comparatively high than national estimate in Bangladesh (8.4%). A nationally representative survey (BDHS 2017–2018) reported that more than 31% of preschool age children are stunted, 22% are underweight and more than 8% are wasted and 2% are severely wasted [38]. The prevalence was heterogeneously distributed across countries: 6.4% in India [39], 10% in Nepal [40], 12.7% in Pakistan [41], 9.5% in Afghanistan [42]. According to the UNICEF, 28.9% of all children were underweight, nearly 40.2% were stunted, 17.7% are wasted, and 28.9% were underweight [43]. This study assumed that, as the area was situated at very near of divisional and educational city and that most of the children living in these areas, all respondents were normal nutrition, but this study demonstrated that a large number of malnourished children were found. Near about age 6–23 in month children were 2.8% suffering severe acute malnutrition similar results were found in capital city in Bangladesh [44], and other population [45]. We observed that more number of girls were severe acute malnourished than boys and similarly more number of girls were moderate malnourished than boys. Our findings supported to another study in Rajshahi city, authors found that the prevalence of malnutrition (under nutrition) of under-five girls was higher than under-five boys in all the anthropometric indicators [46]. Same finding was found in a nationally representative survey in Bangladesh, they reported that the prevalence of malnutrition (under nutrition) among pre-school female children was higher than pre-school male children in Rajshahi division [47]. The difference may be due to discriminatory behavior of the parents. According to Choudhury, gender gap persisted in the society of Bangladesh, with female children was 1.44 times more likely to be severely malnourished [48]. Similar results found in Kenya where girls were more stunted, underweight and wasted than boys at all age categories due to their consistent lower food intake [49].

This study observed that younger preschool children were 2.29 times risk than older children; alongside young mother’s children were more suffering the acute malnutrition compare to more aged mothers. Similar results were found in India and Nepal [50, 51]. In this study we found that children were more acute malnutrition suffers who came from low income family having no hygienic latrine facilities. Similar results were found in other studies in Nepal [52], Ethiopia [53], Nigeria [54], they found that poverty one of the crucial factors for malnutrition among under-five children.

### Strength and limitation of the study

Perhaps this was the first time we attempted to analyze the malnutrition measured by MUAC of pre-school children living in Rajshahi district, Bangladesh. We tried to determine the individual effect of each selected predictors of acute malnutrition. The study could be generalized to other types of children in Bangladesh because children from all sectors have almost similar level of characteristics in Bangladesh. However, the authors acknowledge some limitations. The present study was a cross-sectional quantitative design. The cross-sectional nature of the included studies means causal inferences were not possible. There was a risk of selection bias. Preschool children who were exposed to acute malnutrition or other illnesses might had higher rates of absenteeism from survey and hence more likely to exit missed by the survey. Prospective studies might confirm these results using more objective data such as measured potential indicators by applying robust designs and measurement of diet quality such as longitudinal cohorts and randomised controlled trials analyses (or any appropriate study design). The analyses were based on individual self-reported data (except for anthropometric data) rather than measured data. This had been recognised as a limitation, which might lead to some misclassification in terms of recall and social desirability biases.

## Conclusion

The burden of acute malnutrition (AM) was high among preschool in the regional part of Bangladesh, which is an alarming issue for policy maker. Some factors were associated with AM of preschool children such as (i) children age, (ii) mothers’ age, (iii) family monthly income, (iv) religion and (v) household having hygienic latrine. Girls were more vulnerable for getting acute malnutrition than boys. These findings can help to the health authorities of Bangladesh Government and non-government organizations for improving their health policy to reduce or remove acute malnutrition among children in this country. This study also strongly recommended that advocacy communication for social mobilization, food security and nutrition intervention most urgent for eradicated acute malnutrition from Bangladesh.

## Data Availability

Data is available, and it will be provided by corresponding author when Journal ask.

## Abbreviations

AM: Acute malnutrition
aOR: Adjusted odds ratio
BDHS: Bangladesh Demographic and Health Survey
CC: Community Clinic
CI: Confidence interval
IBM: International Business Machines Corporation
LMICs: Low- and middle-income countries
MAM: Moderate acute malnutrition
MUAC: Mid-upper arm circumference
SAM: Severe acute malnutrition
SDGs: *Sustainable Development Goals*
SE: Standard error
SPSS: Statistical Package For Social Sciences
UP: Upazila
WHO: World Health Organization
WHZ: Weight-for-length or height z-score
